# Technology for nature conservation: An industry perspective

**DOI:** 10.1007/s13280-015-0702-4

**Published:** 2015-10-27

**Authors:** Lucas N. Joppa

**Affiliations:** Microsoft Research, One Microsoft Way, Redmond, WA 98052 USA

**Keywords:** Biodiversity, Collaboration, Cross-sector partnerships, Information age, Nature conservation, Technology

## Abstract

Information age technology has the potential to change the game for conservation by continuously monitoring the pulse of the natural world. Whether or not it will depends on the ability of the conservation sector to build a community of practice, come together to define key technology challenges and work with a wide variety of partners to create, implement, and sustain solutions. I describe why these steps are necessary, outline the latest developments in the field and offer actionable ways forward for conservation agencies, universities, funding bodies, professional societies, and technology corporations to come together to realize the revolution that computational technologies can bring for biodiversity conservation.

## The promise

We live at the intersection of two unprecedented ages. The first is the Information Age of laptops, tablets, smart phones, the internet, social networks, and innumerable miniaturized computing devices which permeate every aspect of daily life (Castells 2011). The second is the Anthropocene (Crutzen 2006; Steffen et al. 2007)—defined by an exceptionally rapid loss of biodiversity caused by human activity and changing climates.

Conservation biology is the scientific discipline that addresses the ‘dynamics and problems of perturbed species, communities, and ecosystems’ (Soulé 1985). The practice of nature conservation has always been interdisciplinary: those dedicated to conserving the ~9 million species on Earth (Mora et al. 2011) are well aware that success often requires efficiently combining ‘mud on boots’ field science in remote areas of the world with the political acumen of a seasoned lobbyist. Now add to that the role of technologist.

The role that computational tools and technology can play in helping monitor, model and respond to the challenges of global biodiversity loss is enormous. I take a broad definition of computational technology here—including the hardware, software, databases, algorithms, and programming languages that come together to turn data into insight. The breadth of this definition is partially out of necessity—in recent years the number of computational approaches to conservation has grown rapidly (Arts et al. 2015).

The conservation community’s embrace of computational technology, and the passion, ingenuity and perseverance that a hugely diverse group of individuals and organizations have brought to this space is immensely inspirational, and the media and public have been paying attention. Stories on drone projects for anti-poaching (Wall 2014), GPS-tagged sharks tweeting their locations to nervous beach-goers (Yu 2014), species rediscovered by remote camera traps (AAP 2014), monitoring of illegal fishing (Craymer 2014), or a crowd-sourced bioblitz (Foderaro 2013) of a local park are a steady feed into the news cycle. With time to ponder the possibilities, a powerful vision appears: Information Age technology changing the game for conservation by continuously monitoring the pulse of the natural world.

## The problem

But there is a persistent concern: for every solidly planned and implemented project (e.g., iNaturalist, eBird—see Wood et al. 2011) there are a host of scattered and inconsistent approaches to using computational technology to solve real conservation problems. The current general approach is a patchwork of one-off projects and partnerships. This wastes time, money, and resources in a discipline that can ill-afford to do so.

Digging beyond the news stories one often finds that the drone has been crippled by a lack of funds and engineering expertise. The new app has a bug—and the intern who wrote it has moved on. The machine-learning algorithm works perfectly on a small dataset—but is missing the infrastructure to scale it beyond the desktop. Camera traps are indeed taking pictures—now the problem is not a lack of images but an avalanche of them (Swinnen et al. 2014). Who, or what, is going to sort through them all? And it turns out that the smartphones used at the bioblitz—and the power and connectivity they require—are not available where ecological surveys are most needed.

These difficulties are partly explained by the different motivations driving the technology and nature conservation domains (Maffey et al. 2015). In technology research the motivations are often academic—proving what is possible and pushing back the research frontiers. Many exciting results emerge, but these mostly end up in published papers, demonstrations or prototypes, after which the researchers move on to the next problem. Technology firms take a few of those results and turn them into products for consumers or enterprise, often losing the features most critical to the conservation community’s needs (like durability, power efficiency, cost, or other important factors). As a result, those working to conserve nature are often inspired by the vision produced by technology research, but left without the tools needed for effective nature conservation.

For example, unmanned aerial vehicles (UAVs or ‘drones’) with sustained flight times in harsh environments, capable of being operated by unskilled workers and performing custom tasks like autonomous monitoring of wildlife poaching via computer vision and acoustic recognition technologies, are still lacking. It is possible to build such an integrated system, but the UAV research community has not seen fit to engineer it (although the Wildlife Conservation UAV Challenge^1^ is working to change that). A wide range of issues—of scale, limited funds, attention, expertise, and unforeseen engineering challenges—needs addressing when adapting computational technology to the needs of nature conservation, a problem not unique to the intersection of conservation and technology (King and Crewe 2013). But these issues of implementation must be overcome in a systematic way if technological approaches are to help, not hinder, conservation practices. This is possible by establishing a common core of required technology and partnering with academic institutions, funding bodies and the private technology sector in a sustainable manner to create a community of practice in conservation technology (see Galán-Díaz et al. 2015 for a cost-benefit evaluation of digital innovation in nature conservation through partnerships working with academics).

## Building a conservation technology community

The International Union for the Conservation of Nature’s (IUCN) Red List combines information from over ten thousand scientists to classify species by the levels of conservation concern attached to them.^2^ This assists decision-making, from student’s choices of scientific inquiry to helping multilateral funding agencies understand the ecological impacts of their investments. While controversial, global prioritization schemes for site-based conservation (Brooks et al. 2006) brought clarity—and focused funding—to a variety of competing interests. Some, like biodiversity hotspots (Myers et al. 2000), identified areas of the world most important for biodiversity conservation. Others, like the Alliance for Zero Extinction,^3^ work synergistically with the IUCN Red List to identify and protect places where Critically Endangered or Endangered species are confined to a single remaining location. Efforts such as these show that nature conservation has a good track record of aligning around shared objectives (Arts et al. 2015).

In a similar manner, there is now an opportunity to define a shared vision for how computational technologies can have maximum impact on conservation practice—before the individual actors in nature conservation have invested considerable resources in replicating what others are already doing, or pursuing potentially non-optimal solutions.

### Conservation agencies and NGOs

Implementation of conservation projects most often falls on government agencies and conservation NGOs. Internal recognition of the role that technology can play in making them more efficient and effective is necessary. There is progress being made on this front within individual NGOs. For example, the World Wildlife Fund’s (WWF) *Wildlife Crime Technology Project* aims to reduce poaching in key conservation areas by combining the use of unmanned aerial vehicles, satellite remote sensing, wildlife and poaching patrol tracking devices, gunshot detectors, and analytical software. Flora & Fauna International’s (FFI) *Conservation Labs* is intended to be an information sharing portal on the most effective use of conservation technologies, and the Zoological Society of London’s (ZSL, in collaboration with University College London and Microsoft Research) *Technology for Nature* initiative aims to connect those in need of technology with those who can provide it. However, when working with limited resources toward a global good it is often counterproductive to work in isolation. Tying these, and other, initiatives together to ensure that their investments are contributing to a platform of solutions desired by the larger conservation community is the next step. Positive examples can be found in the partnership behind the SMART^4^ software platform for monitoring the effectiveness of conservation activities, and the United for Wildlife^5^ consortium of NGOs that has come together to target the illegal wildlife trade. Such a concerted, cross-organizational effort to understand the technologies that are most urgently needed for nature conservation is a necessary first step in building a conservation technology community. There are early movements in this space, with RESOLVE’s recently created Biodiversity and Wildlife Solutions Program leading an unbiased survey of the sector’s needs and desires. Building a framework across institutions for agreeing on core priorities for investment and engagement is an essential next step.

### Universities and funding bodies

Some of the technologies most beneficial to conservation may be directly available from other sectors (e.g., the consumer market), but it is likely that many will not and must be tailored to the problem at hand. Implementing computational technology at the appropriate scale, in remote, un-instrumented environments with non-specialized personnel is challenging. Additional research and engineering will be required to build the solutions that meet the small, but specialized needs of the conservation community. This is a critical role for universities and funding institutions to fill, and will require strong interdisciplinary work between the traditional domains of conservation science (e.g., biological and social sciences) and the engineering, computer science, and statistics departments. This would expose students in the non-computational sciences to computational thinking, and provide all involved with an outlet to innovate in tackling one of the world’s most pressing problems. Interdisciplinary innovation is the purpose of the US National Science Foundation Office of International and Integrative Activities’ *ScienceTechnologyCenters* (STC). Of the 20 past or present STCs, none have focused on the science and technology of biodiversity conservation. Future rounds of consideration for STC funding provide an opportunity for universities to change that.

### Technology industry

The companies building the infrastructure of the Information Age have the potential to be powerful allies of the conservation community. There are examples demonstrating the industry’s desire to be helpful: Microsoft’s partnership with the IUCN Red List (software to map threats to species around the world), Hewlett-Packard’s ‘Earth Insights’ partnership with Conservation International (analytical software for monitoring threatened species), and Google’s financial donations to NGOs via their Global Impact Awards for anti-poaching technology are just a few. But however well intentioned, the business of these corporations is not conservation. Not surprisingly, to date most corporate engagements with nature conservation have been through programs in philanthropy or societal impact.

While useful for raising awareness and funding conservation efforts, these forms of partnership do not typically hinge on what is unique about the technology sector—a drive to innovate through developing new uses of technology for the masses. An important question then is ‘What does nature conservation offer as motivation for the technology industry to get seriously involved in building tools to conserve nature?’ To achieve true breakthroughs, the technology used in conservation has to solve a classic manufacturing trilemma: strong, light, and cheap. It is relatively easy to optimize on any two, but achieving all three together is difficult. Add to that power efficiency, ease of deployment, element-proofing, remote communication, and a host of other requirements in the conservation domain and conservation begins to look like an enormous attraction for corporations to develop new technologies central to their operating strategies.

The ‘internet of things’ is rapidly becoming the ‘internet of everything’ and what better place to tackle some of the hardest problems in that space than in the pursuit of nature conservation? By integrating more fully with the motivational aspects of what drives the technology sector, those working to conserve nature could perhaps benefit from pivoting their messaging away from financial donations for good and toward environmental systems as technology test-beds. Argued successfully, that strategy has the potential to lead to long-term partnerships where the voice of the conservation community is taken seriously in not only the application, but also the design, of future technologies.

Regardless, to ensure that corporate engagement delivers its potential for conservation, the relevant conservation communities should work together to provide guidance on how the private sector could be most effective to the entire domain—offering a unified set of resource needs and projects on which they could or should be engaged. Again, these resources need not be financial—corporations can endorse secondments of scientists, engineers and program managers that could be personally fulfilling for the employee while furthering the knowledge base of the company. Alternatively, corporations and philanthropic organizations could choose to fund a pool of engineering resources to which individuals and institutions from the conservation realm could submit project applications. These are just two potential opportunities to gain access to skill sets that are in short supply in the conservation community, and provide challenging proving grounds where the technology industry can develop tomorrow’s consumer technologies by solving today’s nature conservation problems.

### Communicating priorities, engaging stakeholders, and sharing best practice

Consolidating technology needs for effective nature conservation will require efficient communication and procedures for determining the priorities across the sector. Once agreed, acquiring the resources—whether financial or human—from multiple sources is an essential next step, followed by building solutions and implementing them in the field. Success (ultimately the reversal of biodiversity loss) will only be obtained by the ability to sustain the solutions through time.

Viewed in its entirety the overall task seems daunting and realizing the ideas articulated here will take time. Yet many of the founding structures are already being built, and creating key points of information exchange will help accelerate the construction of a community of practice that combines domain knowledge of nature conservation problems with the acumen to engineer technological solutions. A centralized website that allows individuals and organizations to post their projects, experiences, and difficulties, and find others with relevant experience would begin bringing together what is currently still a hugely decentralized community (see ConservationDrones^6^ for an example of the bringing together of multiple communities within one defined technology area). An initial ‘Grand Challenge’ at the intersection of nature conservation and technology would raise awareness of, and further strengthen, a growing conservation technology community. Finally, the Society for Conservation Biology (SCB) could take an organizing lead by forming a Conservation & Technology Working Group. This group could partner with industry and other professional organizations in the engineering and computational sciences for annual symposia and conferences, and disseminate content through SCB’s multiple publication platforms.

## Moving ahead

Computational technology might revolutionize the practice of conservation by providing the tools and infrastructure to monitor, model, and safeguard biodiversity in entirely new ways. Whether or not it will succeed depends on the ability of individuals working to conserve nature to come together to define key technology challenges and work with a wide variety of partners to create, implement, and sustain solutions. Doing so presents one of the most fascinating challenges of our time—integrating with the infrastructure of the information revolution to avoid the devastating consequences of depleting Earth’s biodiversity.

## Footnotes

1 http://www.wcuavc.com/.

2 http://www.iucnredlist.org/.

3 http://www.zeroextinction.org.

4 http://www.smartconservationsoftware.org.

5 http://www.unitedforwildlife.org/#!/home.

6 http://conservationdrones.org.
